# How to efficiently move towards primary prevention in sporadic AD

**DOI:** 10.1002/alz70859_105951

**Published:** 2025-12-26

**Authors:** Reisa A. Sperling, Rema Raman

**Affiliations:** ^1^ Center for Alzheimer Research and Treatment, Department of Neurology, Brigham and Women’s Hospital, Boston, MA USA; ^2^ Alzheimer's Therapeutic Research Institute, University of Southern California, San Diego, CA USA

## Abstract

**Objective:**

Alzheimer’s disease (AD) prevention trials have evolved rapidly over the past decade, led by secondary prevention trials, such as the A4 Study, targeting the preclinical stages of AD. Ongoing secondary prevention trials aiming to prevent cognitive decline in preclinical AD, including the AHEAD 3‐45 Study and TRAILBLAZER‐ALZ‐3, are now testing FDA approved medications that have demonstrated ability to substantially reduce amyloid burden in later stages of symptomatic AD. Ultimately, we aim to target even earlier in the disease process and prevent the initial accumulation of amyloid and tau pathology, moving closer to primary prevention of AD.

**Methods:**

Given the time lag between the earliest amyloid accumulation and detectable cognitive decline, advancing primary prevention trials will require use of biomarker outcomes. Bridging evidence from secondary prevention trials that can link biomarker and imaging outcomes to cognitive and clinical benefits will be critical to validate these biomarker endpoints for primary prevention. Plasma biomarkers and algorithms employing age, APOE genotype, p‐tau217 and Abeta42/40 can select individuals likely to accumulate amyloid in the future, but additional data are needed to understand longitudinal variability and demonstrate that plasma biomarkers will accurately reflect the prevention of future amyloid “positivity”. Active immunization, intermittent dosing of passive antibodies and oral agents to reduce Abeta/tau are promising candidates for primary prevention on a global scale.

**Results:**

The findings from the AHEAD Study screening process and longitudinal data from the LEARN Study are providing critical data to guide future prevention trial design. The Alzheimer Plasma Extension Study (APEX) is enrolling over 1000 individuals who did not meet amyloid eligibility criteria for AHEAD but showed subtle biomarker abnormalities on plasma testing. APEX is oversampling individuals from underserved racial and ethnic communities to evaluate longitudinal trajectories in AD biomarkers, but also markers of vascular, metabolic and inflammatory processes that may contribute to cognitive decline in diverse communities.

**Conclusions:**

Optimizing clinical trial design is essential for advancing Alzheimer’s prevention on a global scale. Building large international cohorts with “run‐in” plasma biomarker data with global collaborations will serve to accelerate the development and accessibility of Alzheimer’s prevention solutions.

